# Combining Genetic Algorithms and SVM for Breast Cancer Diagnosis Using Infrared Thermography

**DOI:** 10.3390/s21144802

**Published:** 2021-07-14

**Authors:** Roger Resmini, Lincoln Silva, Adriel S. Araujo, Petrucio Medeiros, Débora Muchaluat-Saade, Aura Conci

**Affiliations:** 1 Institute of Exact and Natural Sciences, Federal University of Rondonópolis, Cidade Universitária, Rondonópolis 78736-900, MT, Brazil; roger@ufr.edu.br; 2 Visual Lab, Institute of Computing, Fluminense Federal University, Av. Gal. Milton Tavares de Souza, S/N, Niterói 24210-346, RJ, Brazil; lincoln@lampada.uerj.br (L.S.); aconci@ic.uff.br (A.C.); 3 Advanced Research Medical Laboratory, Departament of Information Technology and Education in Health, Faculty of Medical Sciences, State University of Rio de Janeiro, R. Professor Manuel de Abreu, 444, Rio de Janeiro 20550-170, RJ, Brazil; petruciomedeiros@id.uff.br (P.M.); debora@midiacom.uff.br (D.M.-S.); 4 Mídiacom Lab, Institute of Computing, Fluminense Federal University, R. Passo da Pátria 156, Niterói 24210-240, RJ, Brazil

**Keywords:** breast cancer, diagnosis, thermography, genetic algorithm, support vector machine

## Abstract

Breast cancer is one of the leading causes of mortality globally, but early diagnosis and treatment can increase the cancer survival rate. In this context, thermography is a suitable approach to help early diagnosis due to the temperature difference between cancerous tissues and healthy neighboring tissues. This work proposes an ensemble method for selecting models and features by combining a Genetic Algorithm (GA) and the Support Vector Machine (SVM) classifier to diagnose breast cancer. Our evaluation demonstrates that the approach presents a significant contribution to the early diagnosis of breast cancer, presenting results with 94.79% Area Under the Receiver Operating Characteristic Curve and 97.18% of Accuracy.

## 1. Introduction

Breast cancer is one of the leading causes of mortality globally, and, last year, it victimized around 685,000 women [1]. Projections indicate that around 12.9 million people will die due to cancer by 2030 and around 857,000 caused by breast cancer, according to the Global Cancer Observatory (GLOBOCAN) [2]. These high mortality rates are associated with late diagnosis of the disease. To improve that perspective, affirmative actions to make the population aware have been employed encouraging prevention and early diagnosis. Early detection and treatment improve such prognosis [3].

Breast cancer diagnosis is carried out mainly by imaging examinations. Mammography highlights the formation of denser tissues (calcifications) and has been widely used worldwide to detect breast cancer. However, this examination is not so efficient for dense breasts and presents substantial false positive rates [4]. Moreover, this acquisition could cause discomfort to the patient and uses ionizing radiation (X-Rays), increasing by up to 2% the patient’s chance of developing cancer [5].

Thermography appears as a low-cost alternative, which does not use ionizing radiation, venous access, or any invasive procedure and also can act as a complement to mammography in several scenarios, contributing to the patient’s diagnostic process. This examination considers the temperature variation on skin surface [6] to identify the presence of anomalies. However, human interpretation of those images is complex and, for this reason, artificial intelligence algorithms must be used to help the medical team to identify the differences between sick and healthy breast patterns.

In this work, we propose a methodology for diagnosing breast cancer based on thermal images. Our proposal builds an ensemble method for selecting models and features by combining a Genetic Algorithm and the Support Vector Machine (SVM) classifier. After a few generations of the Genetic Algorithm, this method selects the models that best fit the problem of breast cancer classification. Subsequently, these models are employed in the features selection process, which seeks to find the smallest features that best classify the data. In addition to generating an effective breast cancer diagnosis method, the combination of these strategies optimizes identifying the parameters and features that best adapt and describe the classification problem. We minimized the most demanding task and maximized fine-tuning choosing the most significant features for breast cancer classification.

Furthermore, the proposed methodology is designed to be applied under different data sources. To assess this methodology’s applicability, we evaluate its performance under different perspectives, combinations of databases, segmentation methods, and groups of features. Four experiments are designed, where six sets of features are computed from two different thermographic breast image datasets. In addition, the experiments use different approaches for segmenting the region of interest to generate different data sources.

In the remainder of this paper, Section 2 summarizes some important related work emphasizing their characteristics. Section 3 presents the ensemble and feature reduction approach used in this work. The database, acquisition protocols, and other essential information are presented in Section 4. Section 5 details the core and main contribution of this work: the proposed methodology for the diagnosis of breast cancer. An evaluation of this methodology under different perspectives is presented in Section 6. Section 7 considers experimental analysis and comparison with other work. Finally, in Section 8, conclusions, limitations, and future work are presented.

## 2. Related Work

The first studies using thermography to detect breast cancer were unsuccessful because infrared camera technology was not able to record temperature variation [7]. Technological advances have allowed cameras to become more sensitive, and temperature differences in the breast infrared thermography of patients with tumors have been highlighted [8]. Posteriorly, Connell et al. [9] indicated that thermal images are suitable to aid in breast cancer detection, evaluation of benign conditions, and follow-up procedures. In this context, thermal imaging has become the focus of several studies involving breast cancer detection in recent decades, as shown by Husaini et al. [10]. In this section, we present some of them.

Arora et al. [11] used 94 thermographic images (60 malignant and 34 benign) from the study at New York-Presbyterian Hospital–Cornell (NPH) to detect breast pathology. Their methodology aimed to find areas of higher temperature difference, evaluating thermal asymmetry between breasts after cooling using the Sentinel BreastScan Software. Their results based on Artificial Neural Networks (ANN) achieved a sensitivity of 97% and specificity of 27%. Wishart et al. [12] evaluated temperature differences and thermal symmetry measures using the same software of the previous study to detect patients with breast cancer. They obtained images from a study conducted in the Cambridge Breast Unit—Addenbrooke’s Hospital (CBU). By using artificial intelligence techniques, the authors achieved a sensitivity of 70% and specificity of 48%.

Hossein et al. [13] analyzed 200 thermal images of patients from an experiment that was carried out at Hakim Sabzevari University (HSU) and extracted features of mean, variance, kurtosis, asymmetry, entropy, breast thermal patterns, and difference between breasts and age to diagnose the patient with breast cancer. The authors used a genetic algorithm to select the most significant attributes: difference between breasts, thermal pattern, and kurtosis and obtained a combinatorial model with a 50% sensitivity, 75% specificity, and 70% accuracy.

Krawczyk et al. [14] extracted a series of image features (four basic statistical features, four moment features, eight histogram features, eight cross co-occurrence features, mutual information, and two Fourier descriptors) from 146 thermal images (29 malignant cases and 117 cases were benign) obtained at Brno University of Technology (BUT). The authors used a Multi Classifier System (MCS) to create multiple random subsets of attributes (ensemble). It is worth highlighting that an ensemble consists of a set of independently trained classifiers [15]. The decision tree-based EG2 algorithm was used for classification. This algorithm uses the information cost function (ICF) to select the attribute of each node to build the tree. The ICF is based on information gain and attribute cost. To select the best MCS classifier, they used a genetic algorithm (GA) with a population fixed at 50 individuals, where each individual represents a set of attributes. The crossover was performed at two points. The selection of the new population was performed using an elitist strategy keeping 10% of the best individuals in the population. The GA stopping criterion was the moment when 100 new populations were generated without performance increase (result stabilization). The ensemble has a number of sets ranging from 1, 3, 5, and 7 and uses 10-fold cross-validation. An accuracy of 91.09%, sensitivity of 80.60%, and specificity of 94.82% were achieved with Ensemble V=7.

Gerasimova et al. [16] performed a multifractal analysis over a breast temperature time series, in order to detect differences between tissue with malignant tumor and healthy tissue. The authors noticed that complex scalar multifractal properties over autonomic regulation series were drastically altered in cancerous breasts. In this preliminary study, they considered six women diagnosed with breast cancer and three healthy women acquired at Perm Regional Clinical Hospital (PRCH). In another work, Gerasimova et al. [17] considered a larger sample, confirming previous results [16].

Lashkari et al. [18] proposed an algorithm for cancer classification of thermal images collected from patients with suspected breast cancer referred to Imam Khomeini Hospital (IKH). They extracted 23 features, including statistical, morphological, frequency domain, histogram, and Gray Level Co-occurrence Matrix (GLCM) from segmented right and left breast. They selected the best features by reducing their numbers using the minimum Redundancy and Maximum Relevance, Sequential Forward Selection, Sequential Backward Selection, Sequential Floating Forward Selection, Sequential Floating Backward Selection, and Genetic Algorithm (GA). The authors used as classifiers AdaBoost, SVM, k-Nearest Neighbors (kNN), Naïve Bayes, and probability Neural Network. The best results were obtained for the frontal images with the reduction by GA combined with the AdaBoost classifier, which generated a mean accuracy of 85% and 87% for the left and right breast.

Raghavendra et al. [19] presented a study that allows diagnosing patients using a descriptor based on a histogram of oriented gradients (HOG) with kernel locality preserving projection (KLPP) for feature reduction. The authors used 50 thermal images (25 healthy and 25 with malignant tumor) acquired at the Department of Diagnostic Radiology—Singapore General hospital (SGH). This methodology used Decision Tree, Discriminant, k-Nearest Neighbor and Fuzzy Sugeno, Naive Bayes, SVM, AdaBoost, and Probabilistic Neural Network classifiers. The results indicated that the Decision Tree classifier reached an average accuracy, sensitivity, specificity, and area under curve of 98%, 96.66%, 100%, and 0.98%, respectively.

Santana et al. [20] studied the classification of breast lesions (cysts and benign and malignant lesions) from thermal imaging using Extreme Learning Machine (ELM). They extracted features of texture and shape with moments of Haralick and Zernike and used the features separately and combined. Their results with ELM reached 95.56% using the combination of the features. Posteriorly, Santana et al. [21] analyzed the performance of several classifiers and concluded that ELM presented the best result with accuracy of 73%, sensitivity of 78%, and specificity of 88%.

Based on the study of Santana et al. [20,21], Silva et al. [22] used texture and shape features computed as moments of Haralick and Zernike. The authors reduced the amount of features using GA and Particle Swarm Optimization (PSO). Their result was classified by: Bayes Net, Naïve Bayes, Multilayer Perceptron, SVM, J48, Random Tree, Random Forest, and Extreme Learning Machine (ELM). The result demonstrated that AG presents a slightly better performance compared to PSO. The ELM classifier with a polynomial kernel of exponent 3 achieved better performance with accuracy equal to 88%.

Baffa and Lattari [23], Fernández-Ovies et al. [24], and Tello-Mijares et al. [25] use a Convolutional Neural Network to classify patients into healthy and unhealthy using thermal images from the DMR-IR database [26]. Baffa and Lattari [23] used DIT (for final diagnosis) and SIT (for initial screening of cases) and obtained 98% of average accuracy with DIT images and 95% with SIT images. Fernández-Ovies et al. [24] obtained best performance with Restnet34 and Resnet50, reaching a prediction of 100%. Tello-Mijares et al. [25] compared the performance of CNN with other classifiers (random forest, multilayer perceptron, and Bayes network): CNN achieved 100% accuracy.

Silva et al. [27] used Support-Vector Machine (SVM) to classify patients as healthy or with breast abnormalities. Their first step is the definition of a Region of Interest (ROI) of each sequence of images. From the ROI, a temperature time series was built, and several features were calculated. The main features were automatically selected in the WEKA (Waikato Environment for Knowledge Analysis) tool using the CfsSubsetEva attribute evaluator and the BestFirst research method and then classified by SVM. The authors achieved 100% accuracy and used the DMR-IR database [26].

Sánchez-Ruiz et al. [28] proposed a methodology for classifying the breast as normal and abnormal. The main contribution of their work was the development of an automatic segmentation of ROI. After the segmentation step, they extracted first and second order statistical features by computing histogram and GLCM for each ROI. They used a GA to reduce the number of features and increase the classifier’s performance. The results obtained were: 98% accuracy, 97% sensitivity, and 100% specificity using the Artificial Neural Networks (ANN) classifier.

Mishra and Rath [29] extracted the Gray level Run Length Matrix (GLRLM) and GLCM features to classify patients between healthy and unhealthy. These features were selected using Principal Component Analysis and Autoencoder and applied to SVM, decision tree, random forest, K-NN, linear regression, and fuzzy logic classifiers. Their results demonstrate that random forest with PCA achieved the best performance with an accuracy of 95%.

Table 1 presents a summary of the related work indicating: the year, general goal (screening and diagnosis), image acquisition protocol (SIT and DIT), number of samples in each class: healthy (H), cysts (C), benign tumor (B) and malignant tumor (M), and database used.

## 3. Ensemble and Dimensionality Reduction

Ensemble methods are an important approach in machine learning techniques, and can be presented in the literature under different names, such as committees of learners, mixtures of experts, classifier ensembles, multiple classifier systems, consensus theory, etc. [30]. The main idea behind the ensemble methodology is to construct a set of models by combining them to solve the problem, and, in a traditional approach, a single model is built around the data [31]. According to [32], there are four levels to build an ensemble, varying according to what is manipulated to build the system:

1. Data level: manipulation is done on attribute values. Several sets of instances are created according to the data value and submitted to the estimator;
2. Feature level: manipulation is done on instance attributes. Different sets of features are created and submitted to the estimator;
3. Classifier level: manipulation of classifiers. Different base classifiers are used to compose a final estimator;
4. Model level: the manipulation is performed on the models, selecting which ones will compose the final estimator. Different models that may or may not come from different base classifiers are combined to result in a single estimator, combining the results of each model.

The strategy of manipulating the learning algorithm is also discussed by the authors of [33], who state that, in this technique, the same base classifier can be configured in different ways to classify the same set of instances, thus generating a variety of classification models. The stacking algorithm is an example of this category.

The area of combining classifiers can be further divided into two possible strategies: classifier fusion systems or classifier selecting systems [34]. The result is the final model by a fusion of better options or selecting from among the options. The fusion or selection process represents the last step of the system. In a problem where the end result is a model that stands out over the other models, the best solution is probably a fusion of better models, while selection is more advisable for problems where the winning model has an overall score, which may be by majority vote, significantly superior to that of its competitors.

In the fusion process, it is complicated to bring together the decisions of several models, especially if they are from different base classifiers, and the problem with the selection is that there must be an “oracle” capable of correctly asserting which model is the winner of the group [32]. In this work, we focus on the selection approach. The flow to deal with ensembles using the selection strategy can be divided into two phases: the generation of the set of models and the selection itself.

In generation phase, an important task involves generating a set of mutually complementary classifiers whose combination provides optimal performance. Furthermore, it can be argued that a success set is one whose generated predictors are accurate and which makes mistakes in different parts of the input space. Another important point is that a greater diversity of models positively impacts the system’s results. When models are generated using the same induction algorithm, the set is called homogeneous; otherwise, it is called heterogeneous.

Different models can be experts at different points in the search space (great places for the general problem). Therefore, an appropriate approach is to select the most suitable model or set of models from the group. This approach is known as Dynamic Classifier Selection (DCS), when it selects a single model, or Dynamic Ensemble Selection (DES), when a selection outcomes a set of models (an ensemble). However, the selection of models can be static or dynamic. In the static selection, the selection of models is performed in the training phase and, in the dynamic selection, the selection of models is performed for each new test sample in the testing phase. The dynamic selection has been a preferable solution.

Whichever strategy is chosen, the biggest problem in model selection is in the evaluation strategy of the best model or set to be selected. A usual approach is to use the result of the model in the test phase to compose a ranking and make the choice from this list.

Bucket of models is an ensemble technique in which a model selection algorithm is used to choose the best model from the set. Therefore, a “bucket of models” approach cannot perform better than the best model in the set [35].

A well-known ensemble learning method is stacking. It is a generic framework that combines several ensemble methods and an enhanced extension of bucket of models that supports heterogeneous models in the generation of ensembles [36]. Stacking has two levels of learning: basic learning and meta-learning. In the first one, models are trained with the training dataset. Once trained, models create a new dataset for a meta-model. The meta-model is then trained with this new training dataset. Finally, the trained meta-model is used to classify new instances.

However, defining the best model or set of models is a costly process, especially if the dataset contains many columns (attributes). In addition, many have a strong dependency between the dataset and the defined model. Selecting a subset of attributes with a very small number of samples and a large number of attributes represents a serious challenge. The success of an attempt to select attributes from a large dataset depends on several factors: the underlying probability distributions (some problems can be easy to solve), the sample size (number of instances), the dimensionality ( number of attributes), the method chosen for attribute selection (how well it finds good subsets of attributes, how robust it is for overfitting, how precisely the criterion of interest is evaluated), and the classifier subsequently recommended to the user. Recent resource selection approaches consider evolutionary learning as one possible way to deal with them [37].

## 4. Datasets

We used the thermal images available at the Database for Mastology Research with Infrared Image (DMR-IR) (The Database for Mastology Research with Infrared Image (DMR-IR) is available at http://visual.ic.uff.br/dmi, accessed on 29 June 2021) and the private thermal image database of the Federal University of Pernambuco (UFPE). Thermal images are acquired through a camera sensitive to infrared radiation that varies according to the equipment used. The DMR-IR uses a FLIR SC620 and the UFPE database uses a FLIR S45 camera.

DMR-IR database was approved by the Research Ethical Committee, registered under the number CAAE: 01042812.0.0000.5243 at the Brazilian Ministry of Health platform (Brazilian Ministry of Health platform is available at http://plataformabrasil.saude.gov.br, accessed on 29 June 2021). UFPE database also was registered in the Brazilian Health Ministry and approved by the Ethics Committee of University of Pernambuco (CEP/CCS/UFPE n° 279/05).

### 4.1. DMR-IR

DMR-IR manages and retrieves breast exam information and clinical data from volunteers. Its goal is to support the scientific community in the development and comparison of computational methodologies that assist in the detection and diagnosis of breast cancer. It has SIT and DIT images, but in this work we use only SIT images.

**Figure 1:**
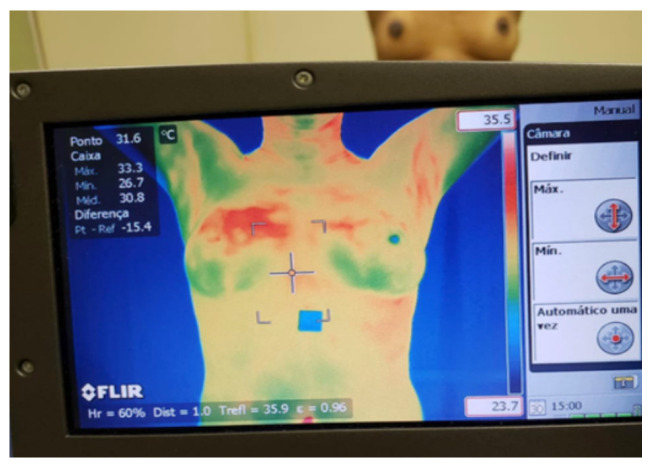
Thermogram acquisition from volunteer [38].

In the static protocol defined in DMR-IR, five (5) thermal images (one frontal and two lateral left and right in the positions of 45° and 90°) are acquired from volunteers standing with hands on their head, after acclimatization for 10 min in a room with the controlled temperature between 20 °C and 22 °C (see Figure 1).

From this database, we selected 80 examinations with 40 “no cancer” and 40 “cancer” diagnosis. The group “no cancer” contains normal and benign cases.

### 4.2. UFPE

The protocol established for the acquisition of thermal images from UFPE uses equipment that allows the volunteer to sit and uphold their arms. At the beginning of the image acquisition protocol, the temperature of the room is recorded and thermal images are acquired after acclimatization for 10 min. The images are acquired from the frontal position with hands on the head and waist, with a metallic grid, internal side of the left and right breasts, and external side of the left and right breasts.

Unlike DMR-IR, the thermal images in this dataset are not publicly available. We selected 98 examinations with 60 “normal” and 38 “cancer” from the UFPE database. An interesting point to note is that the distribution of cases from the UFPE database makes it unbalanced. To deal with unbalanced classes, we used performance evaluation measures that do not privilege the majority class, such as Area Under the Roc Curve (AUC), F1 Score(F1), sensitivity (SENS), and specificity (SPEC) [39]. The goal is to show that the proposed methodology can provide the best classifiers for both, unbalanced and balanced bases.

## 5. Proposed Methodology

This work presents a methodology to diagnose breast cancer based on thermal images using an ensemble strategy. The ensemble strategy proposed in this work is based on a model selection strategy using SVM as a single base classifier. As the oracle of the selection system, we use a ranking of the performance of each model in a dynamic ensemble selection using a Genetic Algorithm capable of generating and selecting the most adapted models. We also built another Genetic Algorithm to perform the attribute selection evaluated by the models resulting from the previous Genetic Algorithm.

The main idea is based on two ensembles, one for model selection and one for attribute selection. The first ensemble aims to select models partially adapted to problems in a few generations of the Genetic Algorithm. This strategy aims to save resources, as, at this stage, all dataset attributes are sent for model training and testing. A set of best models is selected at the end of the process (the best models adapted in a few generations). The next ensemble aims to select the smallest set of attributes that best fit the data. As a fitness function in this step, the set of models selected in the previous step is used. We use another Genetic Algorithm for this step, and this time with more generations looking to achieve the best result by finding the best feature set that generates the best classification result without changing the base classifier configuration. We take into account the dependency between classifier and attributes [37], minimizing the heaviest task and maximizing the fine-tuning that is choosing the most significant attributes for the problem.

**Figure 2:**
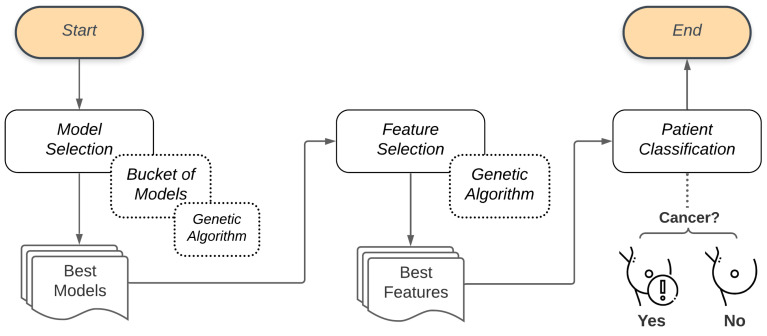
General flow of the proposed methodology.

The proposed system presents three stages. In the first, there is the selection of models, performed by the Bucket of Models technique. The outcome of the Bucket of Models is a set of ten models that do the classification of the problem but are selected after a few generations of the Genetic Algorithm. This set is then taken to the second stage, where another Genetic Algorithm performs the feature selection, testing several sets of features. The outcome of the second Genetic Algorithm is the smaller set of features that best describes the problem. At the end of this process, the resulting model is used to classify the patient (diagnosis). Figure 2 illustrates the general flow presented in this work.

We choose a Genetic Algorithm to perform Bucket of Models and the feature selection because the Genetic Algorithm is indicated to be used in scenarios where there is not much data available for evaluation (the amount of breast thermal images is not huge). In addition, the theoretical analysis (knowledge base) of the problem is also not extremely rich (the use of thermal images is not a consensus among physicians and the analysis is performed visually many times). Thus, we can affirm that data and theory about this problem are poor. Another fact to support this scenario is that the golden standard exam to classify breast cancer is mammography that shows no more than 80% of accuracy in a visual analysis. In other words, the analysis is subjective.

**Figure 3:**
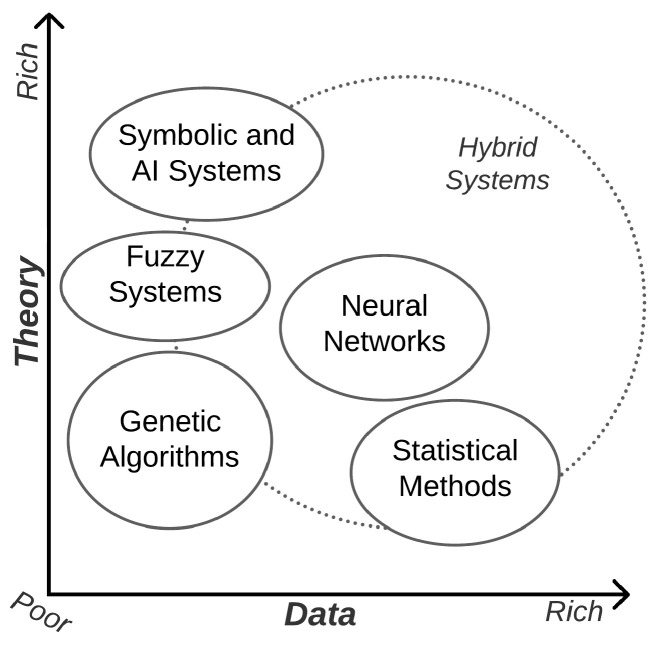
Usability of different methods for problem-solving depending on availability of data and expertise (theories) on a problem [40].

Using these techniques is in line with the Theory of Intelligent Systems [40], illustrated in Figure 3, where the ideal scenario of applying the Genetic Algorithm is positioned in a middle path where there is not much data available and the theory on the problem is not notably high. Alternatives such as Convolutional Neural Networks and Deep Neural Networks, on the other hand, could perform better when working with a larger amount of data.

The proposed methodology is designed to be applied and functional for different particularities of the databases, providing the set of models and features best suited to diagnosing breast cancer. In this work, we evaluated this methodology (Section 6) using two distinct databases that have thermographic breast exams captured under different acquisition protocols. Furthermore, for each database, we investigated the methodology’s performance working under different groups of features computed from distinct regions of interest.

The particularities of the model and feature selection process, as well as the use of the Bucket of Models, Genetic Algorithms, and SVM, are detailed in the next subsections.

### 5.1. Model Selection

Machine learning algorithms are used, among other tasks, to build a model for classification. The construction of this model involves the choice of a basic classifier, which, in turn, can admit a series of parameters that assume different values. Each change in these values results in a new model that fits the data differently and presents different performance values. Thus, defining the best parameters that will fit a model is a search problem.

The most widely used approach to model selection is cross-validation selection, which can be described as “try all models and choose the one that produces the best result” [41]. This approach, however, requires an exhaustive search among all the options of model configurations. Several times, the researcher performs these configurations manually varying such parameters [42]. The choice of the best configuration of a classifier can be made by combining (by selection or fusion) classifiers in several ways. In this sense, we have the techniques of Bagging, Boosting, Staking, and Bucket of Models.

#### Bucket of Models

The Bucket of Models is responsible for providing all possible model configurations from: (a) a base classifier and (b) the set of parameters for such a base classifier, as well as their respective values. Differently from other techniques, the Bucket of Models selects the best fit model from several models (bucket).

**Figure 4:**
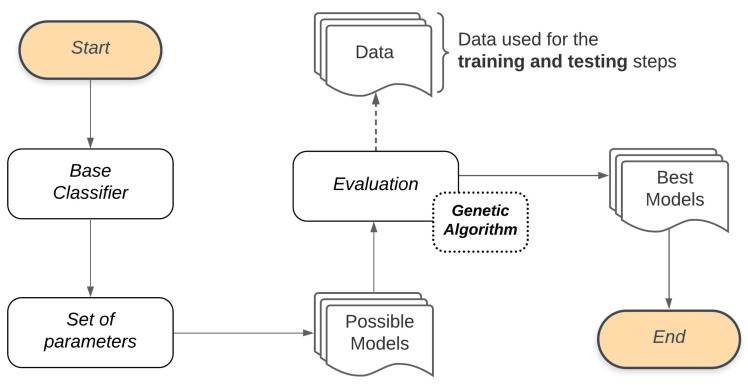
Model selection general flow.

We use a Genetic Algorithm to manage the Bucket of Models. A Genetic Algorithm works generating successive new populations that are a set of individuals, and each individual represents a model. Thus, a Genetic Algorithm provides a Final Set, which in this stage refers to the set of models that best adapt to the classification problem. Figure 4 illustrates the process adopted by the Bucket of Models in our study. Combining the Base Classifier with the Set of parameters will generate the Possible Models to be used in the Evaluation process through the Genetic Algorithm. To evaluate these models, the Genetic Algorithm will also use the set of features that describes the classification problem as input Data. These data are used for training and performance evaluation of each generation of the Genetic Algorithm models. The models resulting from this process will be used in the subsequent step of this methodology (feature selection).

### 5.2. Feature Selection

The outcome from the first step is a solution set with models best adapted to the classification problem. However, these models may not necessarily present the best results. This is the equivalent of stating that the models are great candidates to solve the classification problem.

Therefore, selection of features improves the final result of the classification by evaluating a set of features that best describes the problem. This task maximizes the correctness of the classification since the reduction in the number of features can improve the classification results [33].

**Figure 5:**
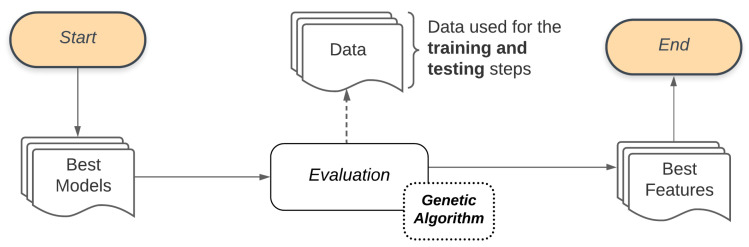
Feature selection general flow.

For this task, we use a Genetic Algorithm where each individual is composed of a sequence of features computed from the images. For each individual, some features are enabled, and others are not. The Genetic Algorithm evaluates, through successive new generations, these individuals and provides the smaller set of features (the Final Set) that best describes the classification problem. For the Evaluation process, in each generation, the Genetic Algorithm for feature selection will also use the set of features that describes the classification problem as input Data. At the end of this process, the ensemble proposed in this study will select the Best Features, the smaller set of features with the best outcome. As many problems could be unbalanced, we define Area Under the ROC Curve as a performance measure. Figure 5 illustrates the flow of feature selection. Figure 5 illustrates the flow of feature selection.

Unlike grid search, the proposed method does not waste time searching in non-promising regions, saving processing resources and computation time. In addition, the algorithm inserts certain randomness into the exploration process, making some searches even in unexplored regions.

### 5.3. Genetic Algorithm

A Genetic Algorithm is an evolutionary algorithm that generates solutions of search problems using techniques inspired by the natural evolution of species [43]. Solutions based on genetic algorithms usually generate several populations of individuals based on evolutionary strategies, such as heredity, mutation, and natural selection [44]. In this work, we used a Genetic Algorithm that combines all these techniques.

As we mentioned before, there are several strategies to evaluate the result of ensembles. We choose to use the classifier itself as the performance evaluator. Thus, the Fitness Function is composed of a model created from the base classifier.

**Figure 6:**
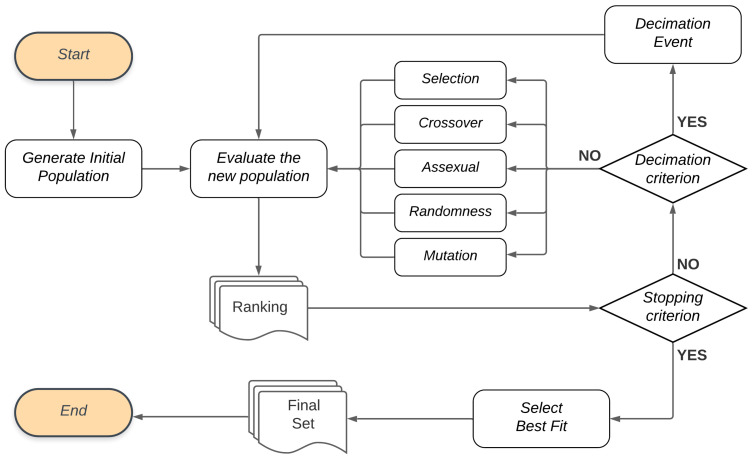
Genetic Algorithm flow.

In addition to this proposal of using GA, this study also considers the occurrence of decimationevents. When these events happen, few individuals survive and the rest are randomly created. In this proposal, such events happen if the solution set has presented the same performance for several generations, and we call this Perturbation. Figure 6 illustrates the general flow of the genetic algorithms proposed in this work.

In the Genetic Algorithm flow, the first population of individuals is randomly generated (“Generate Initial Population” step) and subsequently taken for evaluation. For the Evaluate new population step, the objective function will evaluate each individual in the population, based on a performance measure, and create a Ranking list. If the Stopping criterion is not met, a new population is generated at the end of the evaluation.

If the Stopping criterion is met after the population has been evaluated, the algorithm ends the cycle of creating new generations and Selects the best fit for the Final Set. In the model selection, this set corresponds to the best models, and, in the feature selection, it presents the model with the best set of features in terms of performance.

#### 5.3.1. Model Selection Version

Genetically speaking, what makes an individual different from its ancestors are the evolution perceived in its DNA, composed of a sequence of genes. For the model selection of this work, DNA can be understood as the sequence of parameters that each model assumes. Each of these parameters represents a gene of the individual. Thus, with each new generation, evolution takes place in individuals modifying their genes.

**Figure 7:**
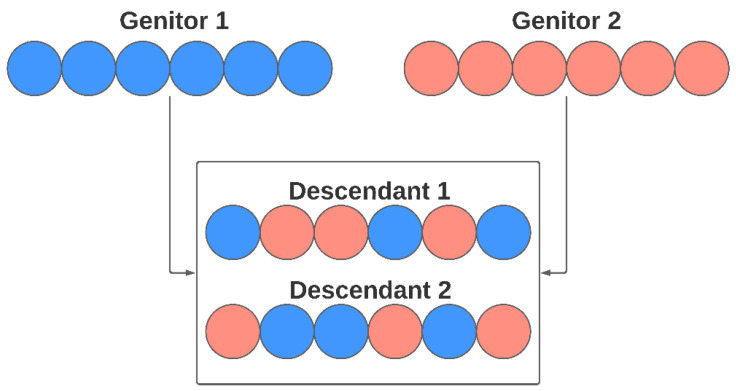
Crossover reproduction in a model selection step.

In the Crossover stage, genes are modified following the concept of heredity, where the genetic load of the new individual is formed from their parents’ genes. In the approach proposed in this work, each descendant receives, at random, genes from both parents. The genetic load is configured to be balanced, receiving the same number of genes from each parent in case of even numbers of them—or almost a similar number of parameters in case of odd numbers of elements. Figure 7 illustrates the crossover process for the genetic algorithm used for the model selection. The number of individuals generated by crossover, in each generation, can be configured by the user.

**Figure 8:**
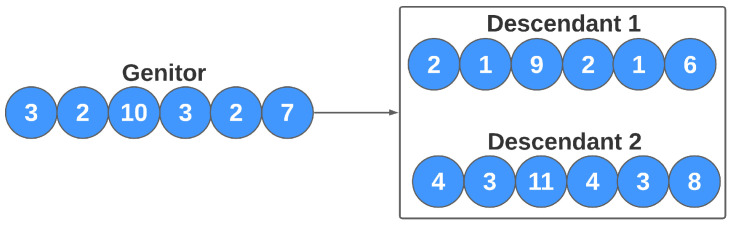
Asexual reproduction for the genetic algorithm.

In asexual reproduction, an individual’s DNA is altered. Thus, this change makes him a new individual in conceptual terms. In this work, this reproduction method refers to the alteration of the values of each model’s parameters (genes). Assuming that a gene *X* has the value *n*, the reproduction will generate two new individuals, where, in one of them, gene *X* has the value n−1, and, in another, it produces the value n+1. This process is repeated for all genes, and represents a fine-tuning of some individuals in the population (the most adapted in the current population) to improve individual performance. In the task of manually adjusting the estimator parameters, the researcher sometimes updates the model with small variations in the parameters. This step tested in our study simulates this behavior. Figure 8 illustrates the flow of the evolution of individuals based on asexual reproduction. In this work, the number of new individuals generated by each of these approaches can also be configured by the user.

Conceptually, the evolution of individuals can also occur by mutation. In the implemented model selection, in each generation, ten individuals undergo mutation: the mutation changes only one gene of the individual to any other acceptable value. However, in each new generation, ten mutations occur, one among the three best, one among the three worst and the other eight occurring randomly in the entire population. The mutation rate is high because it is intended to force the population to high rates of renewal. Unlike creating an individual at random, the mutation works on only one individual’s gene.

Moreover, a new generation may contain randomly generated individuals without necessarily having a genitor. In these cases, in practical terms, the parameters of each new model receive random values (considering the allowed values for each parameter). This randomness helps the solution seek different results that may eventually perform better than the current ones. Consequently, part of the individuals of each generation are formed randomly.

In addition to the reproduction methods, it is also important to mention that individuals considered stronger are selected to compose the new generation. This process is similar to real life, where, in each generation, we have members from previous generations who remain vital for society. This process is only interrupted if a decimation event occurs, where only the strongest individual resists, and all other population members will be randomly generated.

#### 5.3.2. Feature Selection Version

**Figure 9:**
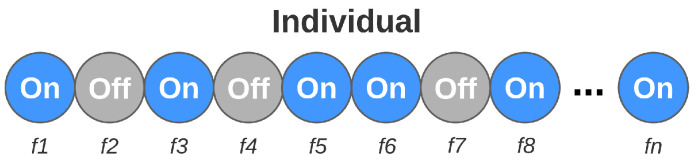
Individual composition in the feature selection step.

The genetic algorithm mechanisms used for feature selection follow premises similar to those previously presented. In each iteration, the strongest members of the population also make up the new generation. In addition, evolution techniques are also considered, modifying the genetic load of another part of individuals. However, as the purpose of this step is to find the set of features that best describes the problem, each individual’s genes now refer to the features themselves. Unlike the previous step, where each gene could assume different values, each gene is binary, assuming only two states: active or inactive. The active genes represent the set of features that the individual is evaluating. The set of active features changes for each individual. Figure 9 illustrates the composition of each individual in the feature selection step.

**Figure 10:**
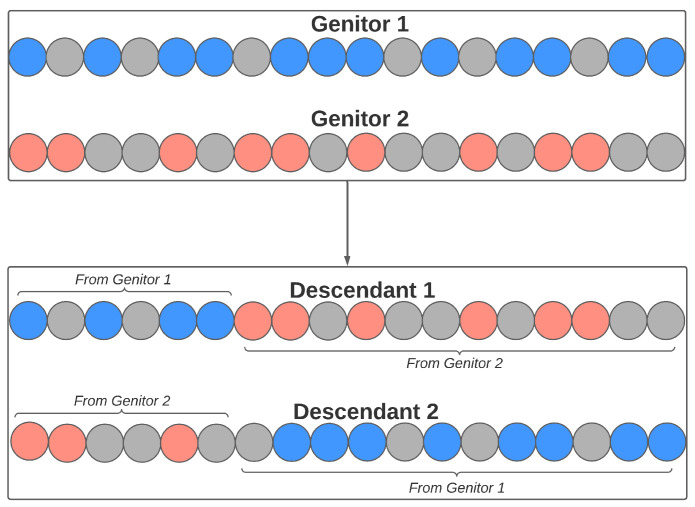
Crossover reproduction in the feature selection step.

Sexual reproduction also occurs from the crossing of two individuals, where each contributes with a genetic part to the formation of their descendants. Each pair of genitors generates two descendants. The number of genes from each genitor is randomly defined, obeying a minimum value (defined by the user). Figure 10 illustrates an example of this process, where the individual has 18 genes. In this example, the cutoff point is 6 (chosen at random), so the descendants of this couple will be composed of the following genetic load: (a) Descendant 1: genitor 1 will contribute to the first six genes in his load; the rest will come from the last genes of genitor 2; (b) Descendant 2: genitor 2 will contribute to the first six genes in his load; the rest will come from the last genes of genitor 1.

As the genes, in this stage of the work, assume binary values, the total number of different potential individuals (considering the complete execution of the genetic algorithm) is smaller. For this reason, the form of asexual reproduction is considered only by mutation (which occurs in a low percentage of the population). As in the previous step, the mutation changes only one gene of the individual (activating or deactivating it). The mutations are programmed to happen at least once in: (a) some of the strongest members (set of features with the best performance); (b) some of the weakest members (set of features with the lowest performance); and (c) any other member of the population.

Random reproduction selects which genes will be activated for each new individual. Devastating events are also considered, where only the strongest member survives, and the rest are randomly generated.

### 5.4. Patient Classification

On completing the model and features selection steps, the ensemble proposed in this work delivers a final model for the classification of breast cancer diagnoses. This model combines the parameters that best adapt to the problem and the best set of features for classifying the images.

## 6. Proposal Evaluation

To consolidate and evaluate all the new aspects proposed in this paper, an evaluation to observe the behavior of the proposed ensembles under the influence of different data inputs was done. For this, different groups of images were considered and, for each of these groups, features based on different computational techniques were computed and applied. Each variation in the input data configures different aspects to evaluate the methodology proposed in classifying breast cancer diagnoses.

### 6.1. Considerations Related to the Databases and the Segmentation Aspect of the Input Images

Two bases of thermal breast images were selected to compose the scope of our evaluation. For each of these bases, two segmentation approaches for obtaining the ROI were used: (a) one in which the evaluated region consists only of the breast area, without considering adjacencies [22,45]; and (b) another one in which the evaluated region extends between the breast and part of the armpits area [20]. This second ROI type aims to consider the lymph node region responsible for transporting the lymph from the mammary tissues to the circulatory system [3]. The combination of two databases and two segmentation methods results in four different configurations of experiments.

For each of these experiments, several sets of features are computed [38]. Each set aims to evaluate, under different data inputs, the behavior of the system under the combinations of classifiers considered in our methodology. Four different approaches to compute features are considered:

1. Gray Level Co-occurrence Matrix (GLCM) [46]:
2. Local Ternary Pattern [47];
3. Daubechies Wavelet [48];
4. Higuchi [49], Petrosian Fractal [50] Dimensions and Hurst Coefficient [51].

In our evaluation, we combined all approaches into six different groups of features. This diversification in several groups allows us to assess the adaptability of the proposed methodology in classifying diagnosis from thermal images based on different features. Table 2 illustrates these groups and the total number of features formed in each one.

### 6.2. Considerations Related to the Input Parameters

We use the SVM classifier [52] as a base classifier for our evaluation because several studies applied to breast cancer for screening or diagnosis have considered this the most adequate classifier for their solution as well [53,54]. This classifier assumes a number of parameters that can be adjusted in order to achieve better performance. In this work, we explore parameters Tolerance, Gamma, Coef0, Nu, Degree, and Kernel types [52], with the following values:

1. Tolerance: [0.1, 0.01, 0.001, 0.0001, 0.00001, 0.000001, 0.0000001, 0.00000001];
2. Gamma and Coef0: [0.00000001, 0.0000001, 0.000001, 0.00001, 0.0001, 0.001, 0.01, 0.1, 0.2, 0.3, 0.4, 0.5, 0.6, 0.7, 0.8, 0.9, 1.0, 1.1, 1.2, 1.3, 1.4, 1.5, 1.6, 1.7, 1.8, 1.9, 2.0, 2.1, 2.2, 2.3, 2.4, 2.5, 2.6, 2.7, 2.8, 2.9, 3.0, 3.1, 3.2, 3.3, 3.4, 3.5, 3.6, 3.7, 3.8, 3.9, 4.0, 4.1, 4.2, 4.3, 4.4, 4.5, 4.6, 4.7, 4.8, 4.9, 5.0, 5.1];
3. Nu: [0.2, 0.3, 0.4, 0.5, 0.6];
4. Degree: [1, 2, 3];
5. Kernel: [’rbf’, ’poly’, ’sigmoid’].

The various combinations between these values will be registered by the Bucket of Models to be used by the Genetic Algorithm to select the models that best adapt to the classification problem.

The ensembles introduced in this work intend to generate good results for diagnosing breast cancer, training a substantially smaller number of models than the search grid, which, as previously mentioned, is considered the most widely used approach for model selection [41]. Thus, the selected range to be explored by the Genetic Algorithm and SVM in the model selection step assume the variations that a search grid would usually practice, varying the values according to the specificities of each parameter [55].

### 6.3. Considerations Related to Reproduction Surroundings

The proposed ensembles allow the user to configure the number of individuals for each approach to population evolution and the total number of generations. In our evaluation, the selection of models will run under 30 generations where, in each generation, the total population will be 140 individuals. The version of the genetic algorithm used for the feature selection will also work with 140 individuals, testing another set of configurations for the evolution of the individuals under 100 generations. The selected number of individuals generated by each reproduction strategy is presented in Table 3.

These configurations are bioinspired. For instance, crossover reproductions tend to be one of the most common forms of reproduction for animals. For this reason, a significant share of the elements of each generation is generated by crossover. In addition, asexual reproductions also occur in different ecosystems, especially those botanic related, thus it also composes the populations of this study. Mutations tend to happen in both fauna and flora, but in a smaller percentage, reflecting on the number of mutations applied in this study. Finally, for vegetables and animals, characteristics from past generations also tend to compose new generations, inspiring the configurations of the proposed method.

### 6.4. Experiment Design

**Figure 11:**
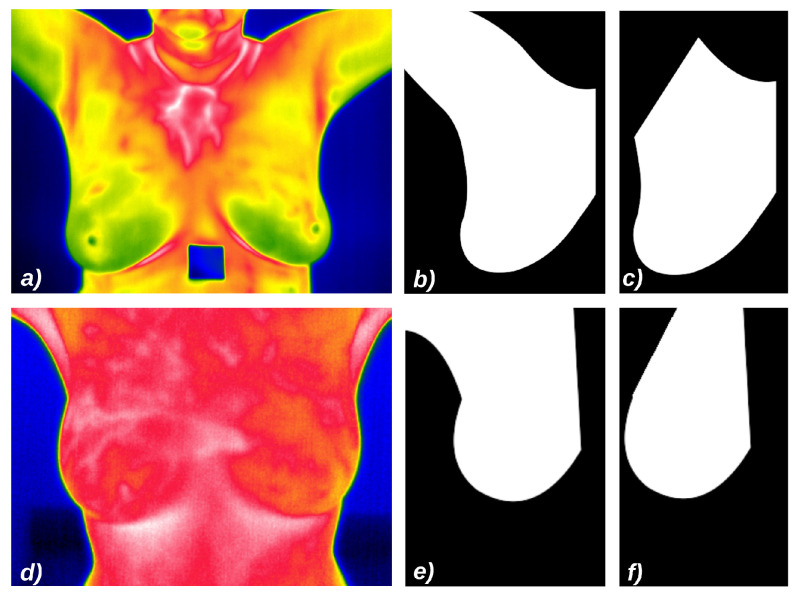
Samples of images from the used datasets, their default false colors and ROIs: (**a**) Image from DMR-IR dataset; (**b**) ROI based on the breast and linfonodes area using DMR-IR images; (**c**) ROI based only on the breast area using DMR-IR images; (**d**) Image from UFPE dataset; (**e**) ROI based on the breast and linfonodes using from UFPE images; (**f**) ROI based only on the breast area using UFPE images.

We ran four experiments to evaluate our proposal:

- Experiment 1: images from the DMR-IR database, and as ROIs the area of breast and armpits as presented in Figure 11b.
- Experiment 2: images from the DMR-IR database, and as ROIs only the breast area, without adjacencies as presented in Figure 11c.
- Experiment 3: images from UFPE database, and as the ROIs the breast with armpits as presented in Figure 11e.
- Experiment 4: images from UFPE database, and as ROIs only the breast area, without adjacent parts as presented in Figure 11f.

For each experiment, the evaluation of every new model, generated in the model selection and the feature selection steps, is performed by the Genetic Algorithm in the Evaluation step. In this phase, each model is trained considering 70% of the data instances and tested, considering the remaining 30% instances, both using the *k*-fold cross-validation where *k*= 4. The details of each database are described in Section 4. For each experiment, all features described in Section 6.1 are computed. Table 4 summarizes these experiments.

## 7. Results and Discussion

The Python programming language, and its libraries and frameworks were used. The feature computation process used methods implemented for this work using the Scikit-Image library, which has a collection of algorithms for analysis and image processing [56]. The model training and testing activities used components from the Scikit-Learn framework, which presents algorithms for machine learning activities in Python [55].

To validate the proposed methodology, the four experiments mentioned in Section 6.4 combining information computed from different areas (ROIs with and without armpits) and using images from different databases, features, and classification approaches were performed as a binary problem, where the instances of features may only have one of two classes: cancer and non-cancer.

### 7.1. Experiment 1

The execution of this experiment had as input the features computed from the thermograms extracted from the DMR-IR. These thermograms were segmented considering the corresponding area between the breast and the armpits as ROI.

Table 5 summarizes the results obtained in this experiment, using AUC as a measure of performance. For each of the groups of features used in this experiment, the performance is presented in two moments: intermediate and final. The intermediate moment represents the best result obtained after completing the model selection. In this step, the genetic algorithm was performed on the various parameter configurations and defined those that best adapt to the classification problem. This result, however, still does not consider the execution of the Genetic Algorithm for feature selection, a crucial step in the proposed methodology. After selecting features, we can see that the final results of the methodology proposed in this study are, for all combinations of features, greater than 80%, reaching up to 96.15% for some groups of features.

These results indicate that the proposed methodology is effective for any group of features considered. In this way, it is possible to observe that this solution seems to be generic enough to deal with different features and presents promising results.

Table 6 shows the outcomes of each group observed from different performance metrics. The results obtained from other performance measures also indicate the robustness of the solution for cancer detection.

### 7.2. Experiment 2

This experiment also used the DMR-IR database as input for the computation of features; however, the regions of interest in this experiment were slightly altered, corresponding only to the breast area, disregarding the possibility of node sentinel observation.

Table 7 illustrates the results of Experiment 2 considering the AUC on model selections and for a final classification of cancer or no cancer. It shows that the proposed methodology remains solid when using the features computed from slightly altered ROIs and with a final reduced range. It is possible to observe that the models generated in the final stage provide good AUC indices, surpassing the indices reached in the intermediate stage of the solution. The results reach good percentages of classification, reaching 94.87% of AUC. In comparison with the ROIs of Experiment 1 (where the armpit region is also considered), the results for some groups of features are slightly inferior; however, they remain satisfying.

The results observed from other performance measures (all of them greater than 86%) also indicate that most patients were adequately diagnosed for the presence or absence of breast cancer, as presented in Table 8.

### 7.3. Experiments 3 and 4

Experiments 3 and 4 use a set of images from the UFPE database. The alteration of the database is interesting because it makes the evaluation of how the methodology behaves in other acquisition protocols and environmental conditions possible, thus analyzing if the results are biased by the used data. The used ROIs (segmented region) of these data also follow two different approaches corresponding to the breast and armpit areas (Experiment 3) and only the breast area (Experiment 4). Table 9 and Table 10 illustrate these results.

Concerning Experiment 3, we can also observe that the diagnoses presented by the classifier combinations proposed in this work reach 97.91% of AUC for final classification, improving the intermediate step of the solution a lot. Similarly, the results of Experiment 4 also behave assertively, evolving between the intermediate and final stages. In both experiments, the diagnostics provided by the solution showed high rates of AUC. Moreover, it is also possible to observe that the other performance metrics also show similar results, with rates above 97%, as seen in Table 11.

### 7.4. Considerations about the Computational Performances

Another contribution of the proposed methodology is the reduction of the processing amount. The selection of features performed substantially reduces the number of features required for diagnosis for all four of the experiments, as shown in Table 12. In practical terms, for future patients to be evaluated, the computation feature process and then the cancer detection will be minimized since we already know which features can better describe the problem.

In terms of performance, it is interesting that the approach proposed in this work provides substantial savings in terms of the number of trained models compared to the exhaustive search. Considering the entire set of possible parameters to be used by the classifier, an exhaustive search would train more than half a million models (precisely 540,560) for each group of features in each one of the experiments in this work.

On using the approach proposed in this work, the model selection stage requires only 0.77% of the total models performed by the exhaustive search, training a total of only 4200 models (140 individuals × 30 generations). Considering the whole solution proposed in this work (model and feature selection), 18,200 models were trained for each group of features (140 individuals × 30 generations for the model selection + 140 individuals × 100 generations for the feature selections). This number represents only 3.37% of all the executions that the exhaustive search would carry out only in selecting models, without considering the feature selection process (crucial for a good classification), on which it would depend on another approach.

### 7.5. Genetic Algorithm vs. Percentile Techniques for Feature Selection

We also compared the result obtained in this study with another technique for feature selection. In this analysis, we use the results from the model selection and, instead of improving them through the selection of features proposed in previous section, we now use the percentile as an approach for such a selection. The percentile is based on univariate statistical tests on the dataset and is also used to select features in works in the literature [57,58].

This comparison is presented in Table 13 and allows us to observe that our genetic proposal leads to better results in practically all the groups. In these cases, considering the six groups of features explored and the four experiments, the results are different in only two of them—even though, in one of them, it can be said that the results are highly similar: 87.5% vs. 86.97%, in the “GLCM + 8LTP” group, on Experiment #4.

### 7.6. Comparison with Related Work

In Section 2, we presented several studies focused on the classification of thermal images involving breast cancer. We can observe that the analyzed studies use combinations of features and classifiers associated with different strategies for screening and diagnosing breast cancer. Moreover, some studies employ complex machine learning techniques like deep learning. We compare our results to those studies and summarize such comparisons in Table 14.

Among related work, we observe that Arora et al. [11], Wishart et al. [12], Hossein et al. [13], Krawczyk et al. [14], Gerasimova et al. [16,17], Raghavendra et al. [19] Santana et al. [20,21], Fernández-Ovies et al. [24], Sánchez-Ruiz et al. [28], and Silva et al. [22,27] do not use the F1-score to assess the classifiers’ performance. On the other hand, only Baffa and Lattari [23] and Tello-Mijares et al. [25] present an F1-score higher than ours, but both use CNN. However, it is worth highlighting that our work diagnoses cancer, while Tello-Mijares et al. [25] do screening. In other words, our work identifies cancer cases (diagnosis task), while Tello-Mijares et al. [25] only identify the presence of abnormalities in the breast (screening task). Furthermore, in general, our performance measures are very close to the performance obtained by Baffa and Lattari [23], except in values of SPEC.

Regarding the ACC measure, Krawczyk et al. [14], Raghavendra et al. [19], Santana et al. [20], Baffa and Lattari [23], Fernández-Ovies et al. [24], Tello-Mijares et al. [25], Sánchez-Ruiz et al. [28], Mishra and Rath [29], and Silva et al. [27] achieve ACC values greater than 90%. Analyzing Santana et al. [20], Fernández-Ovies et al. [24], and Silva et al. [22], we realized that they present ACC equal to 95.56%, 100.0%, and 87.96%, but they use an unbalanced dataset, and the ACC is not a good measure of performance in this case [39].

Examining Krawczyk et al. [14], we observed that SENS is considerably lower than ACC and SPEC, meaning that the study fails to diagnose positive outcomes for patients with breast cancer. Comparing our proposal with the study of Raghavendra et al. [19], we noticed that our results are very close to their performance, and we presented a higher score only in SENS. This result suggests that HOG features can provide a better description of thermal image features.

Arora et al. [11], Wishard et al. [12], Hossein et al. [13], and Gerasimova et al. [16,17] present the worst SPEC measures. Furthermore, comparing our work with Lashkari et al. [18], we realized that our work is robust and presents better results in all the measures analyzed, even without considering the entire amount of features and classifiers analyzed by them. Similarly, Santana et al. [21] present a lower performance compared to ours. Sánchez-Ruiz et al. [28] presented better performance compared to our work, except in the SENS measure, while Mishra and Rath [29] had the opposite response. These results are due to the choice of ANN and Random Forest classifiers.

The results obtained demonstrate that the approach proposed achieves performance very close to deep learning-based approaches, and this work presents a significant contribution to research on diagnosing breast cancer.

## 8. Conclusions

This work introduced a methodology for diagnosing breast cancer based on thermal imaging. For this, two ensemble strategies were proposed. The first one carried out a model selection using a technique called Bucket of Models. To do this, we combined the Genetic Algorithm and SVM to evaluate model results, perform a ranking, and select the best ones in a few generations (30 generations and 140 individuals per population).

The second ensemble method proposed in this work performed feature selection using another Genetic Algorithm, which also worked in conjunction with the SVM. However, the method aimed to obtain the smallest set of features that best classifies the data concerning breast cancer diagnosis at this stage. This step used as input the models best adapted to the problem, resulting from the model selection step, and performed the features selection over 100 generations of 140 individuals each. This task maximized the correctness of the classification since the reduction in the number of features can improve the classification results. Furthermore, we minimized the most demanding task and maximized fine-tuning by choosing the most significant features for the breast cancer classification [37].

We used breast thermal images from two datasets, DMR-IR and UFPE. These images were segmented following different approaches (with and without armpits) to compose four experiments. Armpits host some lymph nodes that have been used to evaluate prognosis results (this technique is called lymph node sentinel). Each experiment evaluates the behavior of the proposed methodology for detecting breast cancer from different images. Six groups of features were computed for the images of each experiment. These features summarize different aspects found in thermographies.

The variety of datasets, segmentation methods, and computed features allow us to assess the robustness of the methodology under different perspectives. In this way, it is possible to observe how much the proposed approaches can learn from the input data and find the sets of models and features that best adapt to the classification problem.

The results achieved by our Genetic Algorithms sequential combination were solid and satisfactory in all experiments, reaching rates of up to 97.91% AUC and 97.18% ACC in the detection of breast cancer. Other indices for quantification of the results also confirm these highest rates for the breast cancer detection process. In comparison with the literature, our work is also powerful and with competitive results on diagnosing breast cancer.

In addition, our proposal optimizes the model training process by more than 96%, compared to the exhaustive search, since the approach does not waste time exploring sets of models and features that do not contribute to the classification process. Through the results obtained by our methodology, it was also possible to observe that it substantially reduces the final set of features that best add value to the classification process—thus minimizing the number of data that need to be preprocessed for classification.

The proposed methodology presented a solid contribution for diagnosing breast cancer using a Genetic Algorithm and Support Vector Machines classifier. This work, however, is subject to limitations that we intend to handle in the next stages of the study, such as: (1) Increase the number of exams used; (2) Investigate the feasibility of using strategies to generate synthetic data; (3) Explore the use of thermographies captured from other angles (e.g., laterals at 45° and 90°); (4) Include data of different breast diagnoses, such as benign tumors and cysts; (5) Explore the applicability of the methodology in more breast thermographic datasets; and (6) Investigate the performance of the methodology under other base classifiers.

Additionally, for the next steps, an evolution of the methodology may assess the difference between the right and left breasts in the anomaly detection process. Finally, the proposed classifier combination system can also be evaluated using datasets, computed from other types of examination, to assess the methodology’s applicability for other open problems.

## Figures and Tables

**Table 1:** Comparison of related work using thermographic images.

Authors	Year	Scre.	Diag.	DIT	SIT	Number of Samples				Database
						H	C	B	M	
Arora et al. [11]	2018	X	X	X	-	34			60	NPH
Wishart et al. [12]	2010	X	X	X	-	41			65	CBU
Hossein et al. [13]	2012	-	X	-	X	185	-	-	15	HSU
Krawczyk et al. [14]	2012	-	X	-	X	-	-	117	29	BTU
Gerasimova et al. [16]	2014	-	X	X	-	3	-	-	6	PRCH
Gerasimova et al. [17]	2014	-	X	X	-	14	-	-	33	PRCH
Lashkari et al. [18] *	2016	-	X	X	-	46	21			IKH (Left Breast)
						34	33			IKH (Right Breast)
Raghavendra et al. [19]	2016	-	X	-	X	25	-	-	25	SGH
Santana et al. [20]	2017	-	X	-	X	-	73	121	76	UFPE
Santana et al. [21]	2018	-	X	-	X	-	219	371	235	UFPE
Baffa and Lattari [23]	2018	X	-	-	X	177	42			DMR-IR
		-	X	X	-	95	-	-	42	
Fernández-Ovies et al. [24]	2019	X	-	-	X	175	41			DMR-IR
Tello-Mijares et al. [25]	2019	X	-	-	X	35	28			DMR-IR
Sánchez-Ruiz et al. [28]	2020	X	-	-	X	142	33			DMR-IR
Mishra and Rath [29]	2020	X	-	X	-	19	37			DMR-IR
Silva et al. [27]	2020	X	-	X	-	32	32			DMR-IR
Silva et al. [22]	2021	-	X	-	X	66	73	121	76	UFPE

* Number of samples from the right (first line) and left (second line) breasts.

**Table 2:** Details related to the description of features.

Description of the Group	N. Features	Detail of the Features
Fractais + Wavelets + 8 LTP	384	3 Fractals × 8 Wavelets × 8 LTP × 2 Breasts
Fractais + 8 LTP	48	3 Fractals × 8 LTP × 2 Breasts
GLCM	48	6 Descriptors × 4 Directions × 2 Breasts
Fractais + 2 LTP + GLCM	60	3 Fractals × 2 LTP Spectrum (0 and 4) × 2 Breasts + GLCM
Fractais + 3 LTP	18	3 Fractals × 3 LTP Spectum (0, 3 and 6) × 2 Breasts
GLCM + 8 LTP	148	100 LTP features + GLCM

**Table 3:** Individuals generated by each reproduction strategy.

	Individuals by	
	Model Selection	Feature Selection
Selection	10	20
Crossover	40	40
Asexual reproduction	20	-
Mutation	10	6
Randon	60	74

**Table 4:** Main elements considered in the experiments.

Experiment	Database	ROI	Features
#1	DMR-IR	Breast + armpits	All features presented in Table 2
#2	DMR-IR	Breast	
#3	UFPE	Breast + armpits	
#4	UFPE	Breast	

**Table 5:** AUC for Experiment 1: Results on model selection and on cancer detection.

Group of Features	*AUC (%)*	
	Intermediary (Model Selection)	Final (Feature Selection)
Fractals + Wavelets + 8 LTP	80.12	**94.87**
Fractals + 8 LTP	80.12	**96.15**
GLCM	80.12	**94.87**
Fractals + 2 LTP + GLCM	75.00	**90.38**
Fractals + 3 LTP	73.71	**83.97**
GLCM + 8 LTP	68.80	**96.15**

**Table 6:** Experiment 1—Final results using various performance measures.

Group of Features	F1 (%)	ACC (%)	SENS (%)	SPEC (%)
Fractals + Wavelets + 8 LTP	94.87	94.61	97.43	93.10
Fractals + 8 LTP	88.46	89.74	91.02	93.10
GLCM	94.35	94.61	94.61	94.87
Fractals + 2 LTP + GLCM	92.16	92.69	92.69	90.38
Fracals + 3 LTP	95.38	95.51	95.51	97.43
GLCM + 8 LTP	95.89	96.15	96.15	97.43

**Table 7:** AUC for Experiment 2: Results on model selection and on cancer detection.

Group of Features	*AUC (%)*	
	Intermediary (Model Selection)	Final (Feature Selection)
Fractals + Wavelets + 8 LTP	81.41	**91.02**
Fractals + 8LTP	81.41	**92.94**
GLCM	83.97	**94.87**
Fractals + 2LTP + GLCM	82.05	**89.10**
Fractals + 3LTP	78.48	**87.82**
GLCM + 8LTP	79.48	**92.30**

**Table 8:** Experiment 2—Final results using various performances.

	F1 (%)	ACC (%)	SENS (%)	SPEC (%)
Fractals + Wavelets + 8 LTP	90.87	91.15	91.15	94.23
Fractals + 8 LTP	92.30	92.69	92.69	97.43
GLCM	94.35	94.61	94.61	94.87
Fractals + 2 LTP + GLCM	88.46	88.84	88.84	91.02
Fracals + 3 LTP	86.15	86.66	86.66	97.43
GLCM + 8 LTP	91.28	91.66	91.66	92.30

**Table 9:** AUC for Experiment 3: Results on model selection and on cancer detection.

Group of Features	*AUC (%)*	
	Intermediary (Model Selection)	Final (Feature Selection)
Fractals + Wavelets + 8 LTP	67.70	**85.41**
Fractals + 8LTP	67.70	**84.37**
GLCM	80.20	**88.54**
Fractals + 2LTP + GLCM	83.85	**97.91**
Fractals + 3LTP	62.50	**85.41**
GLCM + 8LTP	82.29	**97.97**

**Table 10:** AUC for Experiment 4: Results on model selection and on cancer detection.

Group of Features	*AUC (%)*	
	Intermediary (Model Selection)	Final (Feature Selection)
Fractals + Wavelets + 8 LTP	73.43	**90.65**
Fractals + 8 LTP	69.99	**90.10**
GLCM	80.72	**92.70**
Fractals + 2 LTP + GLCM	76.64	**89.06**
Fractals + 3 LTP	83.85	**87.50**
GLCM + 8 LTP	80.20	**86.97**

**Table 11:** Experiments 3 and 4 evaluated by other performance indices.

	*F1*		*ACC*		*SENS*		*SPEC*	
	#3	#4	#3	#4	#3	#4	#3	#4
Fractais + Wavelets + 8 LTP	84.97	90.5	86.77	91.35	86.77	91.35	76.04	85.41
Fractais + 8 LTP	84.55	91.07	87.18	91.87	87.18	91.87	73.95	87.5
GLCM	90.98	91.87	91.77	92.6	91.77	92.6	85.41	85.41
Fractais + 2 LTP + GLCM	97.29	89.01	97.18	90.10	97.18	90.1	94.79	86.45
Fractais + 3 LTP	86.78	85.71	88.54	85.72	88.54	85.72	81.25	81.25
GLCM + 8 LTP	97.29	85.77	97.18	86.04	97.18	86.04	94.70	79.16

**Table 12:** Reduction in the number of features.

	Initial Features	N°. of Features Selected			
		#1	#2	#3	#4
Fractais + Wavelets + 8 LTP	384	18	7	9	45
Fractais + 8 LTP	48	16	23	14	17
GLCM	48	17	17	15	15
Fractais + 2 LTP + GLCM	60	10	7	23	9
Fractais + 3 LTP	18	8	6	7	7
GLCM + 8 LT	148	42	33	22	63

**Table 13:** Comparing Genetic and Percentile techniques for feature selection.

	Experiments							
	#1		#2		#3		#4	
	Perc.	Gene.	Perc.	Gene.	Perc.	Gene.	Perc.	Gene.
Fractais + Wavelets + 8 LTP	70.83	94.87	70.83	91.02	79.16	85.41	90.62	90.65
Fractais + 8 LTP	69.44	96.15	75.00	92.94	79.16	84.37	93.75	90.10
GLCM	81.94	94.87	66.66	94.87	75.00	88.54	90.62	92.70
Fractais + 2 LTP + GLCM	63.88	90.38	50.00	89.10	84.37	97.91	88.54	89.06
Fractais + 3 LTP	63.88	83.97	75.00	87.82	82.29	85.41	82.29	87.50
GLCM + 8 LTP	81.94	96.15	65.27	92.30	90.62	97.91	87.50	86.97

**Table 14:** Proposed method comparison with related work.

Author	F1 (%)	ACC (%)	SENS (%)	SPEC (%)	AUC (%)
Arora et al. [11]	Not informed	Not informed	97.0	27.0	Not informed
Wishart et al. [12]	Not informed	Not informed	78.0	75	Not informed
Hossein et al. [13]	Not informed	70.0	50.0	75.0	Not informed
Krawczyk et al. [14]	Not informed	91.09	80.64	94.82	Not informed
Gerasimova et al. [16,17]	Not informed	Not informed	76.0	86.0	Not informed
Lashkari et al. [18]	68.56	87.42	68.56	92.14	80.35
Raghavendra et al. [19]	Not informed	98.0	96.66	100.0	98.0
Santana et al. [20]	Not informed	95.56	Not informed	Not informed	Not informed
Santana et al. [21]	Not informed	73.38	78.0	88.0	Not informed
Baffa and Lattari [23]	98.0	98.0	97.0	100.0	Not informed
Fernández-Ovies et al. [24]	Not informed	100.0	Not informed	Not informed	Not informed
Tello-Mijares et al. [25]	100.0	100.0	100.0	100.0	100.0
Sánchez-Ruiz et al. [28]	Not informed	98.33	96.67	100.0	Not informed
Mishra and Rath [29]	96.66	95.45	99.17	88.07	Not informed
Silva et al. [27]	Not informed	100.0	100.0	100.0	100.0
Silva et al. [22]	Not informed	87.96	Not informed	Not informed	Not informed
**Proposed methodology**	**97.29**	**97.18**	**97.18**	**94.79**	**97.91**

We consider the measures of greatest values between the screening and diagnosis phases.

## Data Availability

All data used in this research were obtained from two breast thermographic datasets. The first one, the DMR-IR, is completely available at http://visual.ic.uff.br/dmi (accessed on 1 July 2021). The second oh them is a private thermal image database of the Federal University of Pernambuco (UFPE).
